# MicroRNA: an Emerging Therapeutic Target and Intervention Tool

**DOI:** 10.3390/ijms9060978

**Published:** 2008-06-13

**Authors:** Zhen Liu, Alhousseynou Sall, Decheng Yang

**Affiliations:** Department of Pathology and Laboratory Medicine, University of British Columbia, The Heart and Lung Institute, St. Paul’s Hospital, Vancouver, Canada

**Keywords:** miRNA, siRNA, gene silencing, virus, RNAi, heart disease

## Abstract

MicroRNAs (miRNAs) are a class of short non-coding RNAs with posttranscriptional regulatory functions. To date, more than 600 human miRNAs have been experimentally identified, and estimated to regulate more than one third of cellular messenger RNAs. Accumulating evidence has linked the dysregulated expression patterns of miRNAs to a variety of diseases, such as cancer, neurodegenerative diseases, cardiovascular diseases and viral infections. MiRNAs provide its particular layer of network for gene regulation, thus possessing the great potential both as a novel class of therapeutic targets and as a powerful intervention tool. In this regard, synthetic RNAs that contain the binding sites of miRNA have been shown to work as a “decoy” or “miRNA sponge” to inhibit the function of specific miRNAs. On the other hand, miRNA expression vectors have been used to restore or overexpress specific miRNAs to achieve a long-term effect. Further, double-stranded miRNA mimetics for transient replacement have been experimentally validated. Endogenous precursor miRNAs have also been used as scaffolds for the induction of RNA interference. This article reviews the recent progress on this emerging technology as a powerful tool for gene regulation studies and particularly as a rationale strategy for design of therapeutics.

## 1. Introduction

MicroRNAs (miRNAs) are a large family of short non-coding RNAs involved in post-transcriptional gene silencing. They have been found in a variety of organisms including viruses [1, 2]. In animals, miRNAs hybridize to partially complementary binding sites typically localized in the 3’ untranslated regions (3’UTR) of target mRNAs and repress their expression. Efficient repression is either achieved by interfering with translation or by guiding processes for mRNA degradation that are initiated by deadenylation and decapping of the mRNAs [3]. In plants, and in rare cases of animals, mRNAs contain highly complementary miRNA-binding sites and therefore miRNAs guide the sequence-specific cleavage of the mRNA in a process similar to that mediated by short interfering RNAs (siRNAs) [4].

At the present time, although the biological functions of miRNAs are not completely revealed, a growing body of evidence implicates that miRNA pathway is a new mechanism of gene regulation in both normal and diseased conditions and therefore investigation of miRNA biogenesis and function may add new tools for gene functional study and drug development. In this article, we will briefly review the structure, biogenesis and basic mechanism of action of miRNAs identified in higher organisms and viruses and then focus on the recent progress in research for drug development using the miRNA strategy. In addition, we also briefly discuss the certain drawbacks, challenges and future directions on drug development using miRNA as a target or a gene silencing tool.

## 2. miRNA Biogenesis and Mechanisms of Action

In human, the majority of miRNAs (70%) are transcribed from introns and/or exons, and approximately 30% are located in intergenic regions [1]. In human and animal, miRNAs are usually transcribed by RNA polymerase II [5], and in some cases by pol III [6]. Certain viral encoded miRNAs are transcribed by RNA polymerase III [2, 7], and some are located in the open reading frame of viral gene [2, 8]. MiRNA transcription results in the production of large monocistronic, bicistronic or polycistronic primary transcripts (pri-miRNAs). A single pri-miRNA may range from approximately 200 nucleotides (nt) to several kilobases (kb) in length and have both a 5’ 7-methylguanosine (m7) caps and a 3’ poly (A) tail. Characteristically, the mature miRNA sequences are localized to regions of imperfect stem-loop sequences within the pri-miRNAs [9].

**Figure 1.**
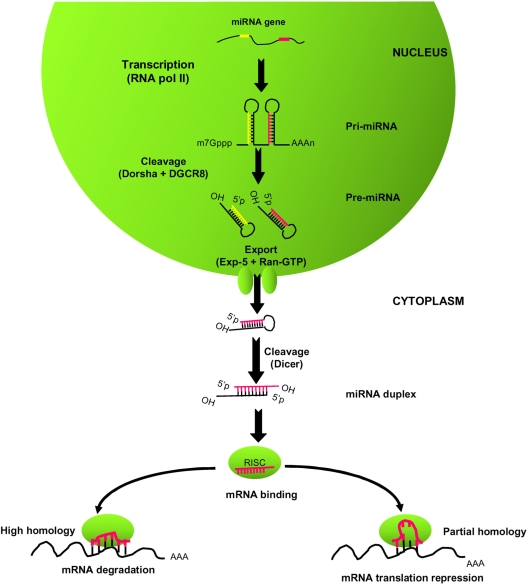
**Schematic overview of biogenesis and function of miRNAs.** The monocistronic or polycistronic miRNA-coding genes are transcribed by RNA polymerase II (Pol II) into long primary transcript of miRNA (pri-miRNA) containing the hairpin structure. These pri-miRNAs are subsequently processed into precursors of miRNAs (pre-miRNA) by nuclear RNase III Drosha with the aid of cofactor DGCR8. Following nuclear processing, the pre-miRNAs are exported to the cytoplasm by a nuclear transport receptor, Exportin-5, and Ran-GTP before being processed by the cytoplasmic RNase III Dicer into 21-nuleotide mature miRNAs. Finally, the single-stranded miRNA are incorporated into an RNA-induced silencing complex (RISC) to induce translation suppression or degradation of viral or cellular mRNAs depending on the degree of complementary with the target mRNA.

The first step of miRNA maturation in the nucleus, as shown in Figure 1, is the recognition and cleavage of the pri-miRNAs by the RNase III *Drosha-DGCR8* nuclear microprocessor complex, which releases a ~70 nt hairpin-containing precursor molecule called pre-miRNAs, with a monophosphate at the 5’ terminus and a 2-nt overhang with a hydroxyl group at the 3’ terminus [10–12]. The next step is the nuclear transport of the pre-miRNAs out of the nucleus into the cytoplasm by *Exportin-5*, a carrier protein [13, 14]. *Exportin-5* and the GTP-bound form of its cofactor *Ran* together recognize and bind the 2-nt 3’ overhang and the adjacent stem that are characteristics of pre-miRNA [15, 16]. In the cytoplasm, GTP hydrolysis results in release of the pre-miRNAs, which is then processed by a cellular endonuclease III enzyme *Dicer* [14]. *Dicer* has been first recognized for its role in generating siRNAs that mediate RNA interference (RNAi). *Dicer* acts in concert with its cofactors TRBP (Transactivating region binding protein) [17] and PACT (interferon-inducible double strand-RNA-dependant protein kinase activator) [18]. These enzymes bind at the 3’ 2-nt overhang at the base of the pre-miRNA hairpin and remove the terminal loop, yielding an approximately 21-nt miRNA duplex intermediate with both termini having 5’monophosphates, 3’ 2-nt overhangs and 3’ hydroxyl groups. The miRNA guide strand, the 5’terminus of which is energetically less stable, is then selected for incorporation into the *RISC* (RNA-induced silencing complex), while the ’passenger’ strand is released and degraded [19, 20]. The composition of *RISC* remains incompletely defined, but a key component is a member of the Argonaute (Ago) protein family [19, 21].

The mature miRNA then directs *RISC* to complementary mRNA species. If the target mRNA has perfect complementarity to the miRNA-armed *RISC*, the mRNA will be cleaved and degraded [22, 23]. But as the most common situation in mammalian cells, the miRNAs targets mRNAs with imperfect complementarity and suppress their translation, resulting in reduced expression of the corresponding proteins [24, 25]. The 5′ region of the miRNA, especially the match between miRNA and target sequence at nt 2–7 or 8 of miRNA, which is called seed region, is essentially important for miRNA targeting, and this seed match has also become a key principle widely used in computer prediction of the miRNA targeting [26, 27]. MiRNA regulation of the miRNA-mRNA duplexes is mediated mainly through multiple complementary sites in the 3′UTRs, but there are many exceptions. miRNAs may also bind the 5′ UTR and/or the coding region of mRNAs, resulting in a similar outcome [28].

## 3. Association of miRNA with Diseases

More than six hundreds of miRNAs have been identified in human but currently, only a handful of specific targets have been experimentally validated. As predicted by bioinformatical analysis, a single miRNA can potentially target different mRNAs through imperfect base complementarity [27]. The high sequence conservation of many miRNAs among distantly related organisms suggests strong evolutionary pressure and participation in important physiological processes by miRNAs. Indeed, miRNA deficiencies or excesses have been correlated with a variety of clinically important diseases including myocardial infarction, virus infection, Alzheimer’s disease, metabolic diseases, cancers, and many others [29–33].

The initial evidence that miRNAs play a collaborating role in cancer came from a study on chronic lymphocytic leukemia [34]. Calin and colleagues found that a region containing miR-15 and miR-16 at chromosome 13q14 was frequently deleted in the majority of chronic lymphocytic leukemia cases [34]. From several studies, it is now clear that many miRNAs are associated with primary human tumors [35, 36], and more than 50% of human miRNAs genes are located at genomic regions implicated in cancers, such as common breakpoint regions and fragile sites [35]. In addition, among three cancer types, i.e. ovarian cancer, breast cancer and melanoma, analysis of the DNA copy number of genomic regions containing miRNAs identified 26 miRNAs with a gain of gene copy number and 15 miRNAs with a loss [37]. It is obvious that miRNAs located in deleted or amplified regions in cancer samples have altered expression levels. For instance, the mir-17-92 miRNA cluster, which is in a region on human chromosome 13, is frequently amplified in B-cell lymphomas [38]. Overexpression of the mir-17-92 cluster was found to co-operate with Myc to accelerate tumor development in a mouse B-cell lymphoma model [39]. Ovarian tumors exhibited gains in DNA copy number of Dicer and Argonaute 2 [37], suggesting that indirect effects could also account for altered miRNA expression in tumors.

Some miRNAs are upregulated in tumors versus normal tissues and thus may act as oncogenes [40, 41], whereas some other miRNAs can act as tumor suppressors. Reduced expression of the former or gained expression of the latter would confer a growth advantage to cells. For example, the miR-17-92 cluster is overexpressed in B cell lymphoma [38, 42], miR-15a and miR-16-1 exert tumor suppressor effect by inhibiting Bcl-2 function [43], whereas miR-155 is oncogenic through its probable effects on transcription factors C/EBPβ and PU.1 [44].

By measuring the expression of human miRNAs in cancer samples, Lu and Volinia et al found that the pattern of miRNA expression varies dramatically across tumor types. Although miR-15a and miR-16-1 are down-regulated in chronic lymphocytic leukemia, which is consistent with their postulated tumor suppressor function [45], the same miRNAs are paradoxically overexpressed in endocrine pancreatic tumors [46]. As might be expected from the role of some miRNAs in development, the miRNA profiles of tumors are in accordance with the tumors’ developmental history. In other words, tumors derived from tissues with a common embryonic precursor share similar miRNA expression patterns. Other miRNA profiling studies have also substantiated malignancy-specific expression patterns in lung [47], colon [48], breast [49] and heptacellular cancers [50]. A recent study has added indirect support that miRNA changes are causal, rather than consequential, of cellular transformation [51]. These suggest that changes of miRNA expression profile might be very informative for cancer diagnosis [47, 52]. The function of individual miRNAs in a variety of cancers has been well reviewed by several research groups [30, 52, 53]. The perturbed expression of these miRNAs during oncogenesis may promote or hamper the tumour formation by modulating the expression of critical genes involved in cancer cell proliferation or survival.

MiRNAs are becoming increasingly recognized as important regulators of heart function. It has been shown that expression of many miRNAs is altered in heart disease and that different types of heart disease are associated with distinct changes in miRNA expression [54]. Thomas et al has reported profound alterations of miRNA expression in failing hearts [55]. Such changes closely resemble the miRNA expression patterns observed in fetal cardiac tissue, suggesting a novel mode of regulation for the transcriptional changes in cardiac failure. Both *in vitro* experiments using cultured cardiomyocytes and *in vivo* studies using miRNA transgenic or knockdown mouse hearts have shown that a subset of these miRNAs is sufficient to drive heart hypertrophic growth and myocyte disarray [29, 56, 57]. The most extensively studied such critical miRNAs are miR-1 and miR-133.

MiRNA regulation has also been implicated in virus-induced diseases. On one hand, cellular miRNA expression may confer host immunity against viral infections; on the other hand, viruses may have evolved to utilize miRNA machinery for their replication advantage. The example of the former came from the study of retroviruses primate foamy virus (PFV) in the human embryonic kidney cell line 293T, which showed that human miR-32 inhibits PFV replication by impairing the translation of viral mRNAs bearing target sequences [58]. The striking example for the latter was from the investigation of hepatitis C virus (HCV) infection, which revealed a smart strategy of this virus to utilize host liver-specific miR-122 targeting the 5’UTR of the viral genome as a positive regulator of viral replication, putatively via conformational change of 5’UTR [59]. In addition, some large DNA viruses of the herpesvirus family, including EBV [2] and SV40 [31], encode viral miRNAs, which may act to modulate host immune response and virus latency.

## 4. miRNA as Therapeutic Target and Tool

In analogy to transcription factors but with opposing suppressive effect, miRNAs provide a particular layer of network for gene regulation of a broad spectrum of biological pathways through fine-tuning protein expression levels and dampening stochastic perturbations of expressed genes. As aforementioned, aberrant cellular miRNAs expression is causally associated with a variety of diseases, and the virus-encoded miRNAs can modulate host immune responses and benefit viral pathogenicity. This implies that miRNAs have great potential to be developed as a novel class of therapeutic targets. To this end, methods for altering the levels of miRNA expression have been adapted from existing gene therapy and RNAi technologies, where synthetic siRNAs molecules have been routinely used to inactivate gene expression in mammalian cells. Specific knockdown of the target miRNAs by anti-miRNA oligonucleotides, or overexpression of miRNA duplex by transfection of vectors encoding this structure has been conducted both *in vitro* and *in vivo* (see later discussion). Although these researches are presently at the bench level, they are promising to be translated into therapeutic agents in the future for tackling diseases on the cellular miRNA level.

In addition, although siRNAs or shRNAs have been widely used as the gene-silencing molecules, and even a few siRNAs are starting to enter clinical trials [60], the intrinsic drawbacks of siRNA methodology have been revealed, including the off-target effects, elicitation of the interferon response, and interference of the endogenous miRNA biogenesis [61–64], all of which greatly hamper its therapeutic use. However, compared to siRNA methodology, the unique biogenesis and mechanism of miRNA action allow it to be exempt from these problems. With the advantage on specificity and toxicity, and the attractive feature of multiple targeting potential, miRNA will also become a great tool for gene intervention.

### 4.1 Antisense inhibition of mature miRNA

Modified anti-miRNA oligonucleotides (AMOs), also designated as “antagomirs”, are currently the most readily available tools for miRNA inhibition [65–69]. As an approach firstly adopted by Boutla and colleagues [70], different types of modified AMOs complementary to mature miRNAs have shown success of inhibiting specific endogenous miRNAs in cell culture [67–69, 71], flies [68, 70, 72], and mice [68, 73, 74]. Three major types of these AMOs are oligonucleotides with the modified 2-OH residues of the ribose by 2′-O-methyl (2′-OMe), 2′-O-methoxyethyl (2′-MOE) and locked nucleic acid (LNA) respectively, as well as additional phosphorothioate backbone modification. These modifications were grafted from those already applied in siRNA methodology. And they are designated to provide nuclease resistance, which is essential for a nucleic acid exposed to abundant serum and cellular nucleases. Another optional modification in these molecules is cholesteryl functionality at the 3’ end of the nucleic acid, to improve the pharmacokinetic properties such as the half-life in serum by its binding to serum proteins and enhanced cellular uptake [75, 76]. In contrast, unmodified 2-deoxy oligonucleotides [77] and 2-deoxy phosphorothioate oligonucleotides [67] were unable to inhibit miRNA activity in cultured cells. It is not clear what the mechanism of miRNA reduction might be [64]. Several reports suggest that anti-miRs interfere with miRNA-mediated silencing by binding to the mature miRNA guide strand in the RISC complex [67, 68, 74]. However, the binding of these AMOs to RISC does not support Ago2 cleavage of target RNA [78, 79]. The degradation of miRNAs results in accelerated turnover of the miRNA complex [80].

In several pioneer studies, the efficiency and significance of various AMOs have been examined and validated by the model that ablates the liver-enriched miR-122 in living mice upon administering the specially developed AMOs [67, 73, 81, 82]. miR-122 is the highly expressed dominant miRNA in the liver, and is implicated in fatty acid and cholesterol metabolism as well as hepatitis C viral replication [59, 79]. Krutzfeldt and coworkers showed for the first time the long-lasting and non-toxic silencing generated by intravenously injected ‘antagomirs’ (2′-OMe) complementary to miR-122 in mice [81]. Unexpectedly, it not only inhibited miR-122, but also led to the degradation of the corresponding miR-122 in the liver and other tissues except central nervous system (CNS), but not the other species of miRNAs in those tissues. This is presumably due to the restriction of the blood–brain barrier. Its durable effect extends as long as 3 weeks. After three days of daily 80 mg/kg antagomirs to normal mice, levels of mRNAs of hundreds of genes containing miR-122 targeting sequence in their 3’ UTRs were increased by a factor of at least 1.4, and a down-regulation of nearly as many mRNAs occurred possibly through the suppression of a transcriptional repressor. Accompanied with the mRNA level of genes involved in cholesterol biosynthesis was the 40% reduction of plasma cholesterol. In a separate article, the same authors found that longer antagomirs (25 nucleotides) were more effective *in vivo* [74], which is presumably due to the higher biostability of the miRNA–antagomir duplex. In addition, a recent study showed that 2′-O-methyl-modified ASOs flanked by hairpin sequences gave enhanced inhibition of miRNAs in cell culture [83].

In another study, Esau *et al*. achieved the same results by delivering intraperitoneally 2′-MOE AMO to silence the miR-122 in mice. The effective inhibition of miR-122 in this study also resulted in a reduction in cholesterol levels and a decrease in hepatic fatty acid and cholesterol synthesis rates, both in normal mice and in diet-induced obese mice, with a significant improvement in liver steatosis of diet-induced obese mice [67, 73]. Thus, it further highlighted the potential of targeting miR-122 with antagomirs for inhibition of metabolic disease development. Moreover, the “second generation” of 2′-MOE has come into being, which differs from former 2′-MOE AMOs only by the presence of a short stretch (8–14 nucleotides) of centrally located 2’ deoxy residues that confers RNase H sensitivity [84, 85].

The third type of AMOs, Locked nucleic acid (LNA), are oligonucleotides that contain one or more nucleotide building blocks in which an extra methylene bridge fixes the ribose moiety either in the C3′-endo or C2′-endo conformation. This modification has provided the oligonucleotides with superior affinity, mismatch discrimination, low toxicity, and increased metabolic stability [86]. An *in vivo* study exhibits LNA as efficient antigomirs, systemically administered 16-nt, unconjugated LNA-antimiR oligonucleotides complementary to the 5′ end of miR-122 led to specific, dose-dependent silencing of miR-122 and showed no hepatotoxicity in mice [82]. Low cholesterol phenotype was also achieved in this study [82]. Further improvement on inhibitory efficiency and cellular uptake was accomplished by a LNA/2′-O-methyl mixmer or electroporation of a peptide nucleic acid (PNA) oligomer, compared to standard 2′-OMe [62].

Nevertheless, effective delivery into target tissues remains a major hurdle for RNAi therapy including the applications of antagomirs and synthetic miRNA duplexes (see section 3.3), before they can be tried clinically [65]. One strategy is to deliver synthetic small RNA molecules by complexing or covalently linking with lipids and/or delivery proteins [82, 87]. Besides nonspecific agent like cationic liposome and cholesterol [88, 89], novel nanotechnology-based conjungation of bacterial phage packaging RNA with therapeutic molecules, receptor binding RNA aptamers, antibody-protamine, etc, have been designed to deliver small RNA assembly to target cells [88, 90]. Conjugation with homing signals for tissue/cell-type specific delivery has recently progressed [88, 91]. All development in this aspect plus the research on local delivery [80, 92–94] may eventually substantiate and expand the therapeutic opportunities for miRNA targeting.

### 4.2 Other strategies for miRNA inhibition

In addition to direct targeting the mature miRNA with ASOs, an alternative way for inhibiting miRNA is to down-regulate components of the miRNA biogenesis pathway, thereby reducing mature miRNA levels [65]. This could be achieved globally by inhibiting *Drosha*, *Dicer*, or other miRNA pathway components, which could be amenable to traditional siRNA silencing [11, 13, 95]. However, to minimize the pleiotropic effects of this approach, tetracycline-inducible shRNA to down-regulate these component expression has been proposed to put it in a highly controlled manner [96]. But in view of the lethal problem that it may cause, the global inhibiting approach may only be used as a research tool.

Currently, there is no RNAi silencing method available for stable inhibition of specific miRNAs because the mature miRNA in *RISC* complexes and premiRNA with a stem-loop are not easily accessible to an siRNA and pri-miRNAs localize to the nucleus, away from the cytoplasmic siRNA. Although in one study [97], siRNA against the loop region of the precursor of a miRNA has shown some potential to downregulate the mature miRNA, the utility of this design has not been further validated, and also the fact that the loop regions of different pre-miRNAs are not conserved may limit its application. Sinuous approaches have been proposed to utilize the putative nuclear form of *RISC* [98], including transcribing two independent strands that can form an siRNA duplex in the nucleus [99] and siRNA-mediated transcriptional silencing by targeting pri-miRNA promoters [100], but they are not very likely to become widely practical approaches for stable inhibition in near future. How, then, can a given cellular miRNA be long-term suppressed?

By adopting the principle of miRNA competitive inhibitors, and possibly inspired by the reporter assay method which are routinely used in identification of miRNA target, Ebert et al [101] developed miRNA inhibitory transgenes expressing an mRNA containing multiple tandem binding sites for an endogenous miRNA. They termed these RNA decoys as ‘microRNA sponges’, which are able to stably interact with or ‘soak up’ the corresponding miRNA and prevent its association with its endogenous targets. Both designed polymerase Pol II- and Pol III-driven miRNA sponges showed more efficiency for miRNA inhibition compared to standard 2′-OMe antigomirs. By introducing a bulge at position of 9–12 in analogy to natural miRNA binding rather than perfect complementarity, these small molecules achieved stronger derepressive effect, possibly due to prevention of mRNA cleavage and increased retention of miRNAs [101]. A remarkable feature of this approach is that miRNA sponge is effective against all closely related miRNAs within a family. This can be particularly useful when members of a miRNA family with overlapping and redundant targets are required to be suppressed. Besides transient and stable knockdown in cell culture, the results of this study also support the feasibility of the generation of transgenic animals expressing inducible miRNA sponges [101].

Previously, an mRNA decoy under the same principle has been designed and applied in the research of miR-133 in the pathogenesis of cardiac hypertrophy [29]. In this study, mice were infected with an adenoviral vector in which a 3′ UTR with tandem sequences complementary to mouse miR-133 and linked to the enhanced green fluorescent protein (EGFP) reporter gene. The complementary sequences act as a decoy, sequestering endogenous miR-133. This suppression of miR-133 by these ‘decoy’ sequences induced cardiac hypertrophy, which was more pronounced than that induced with conventional inducers of hypertrophy.

Appropriate usage of the principle of this approach in gene therapy research has enhanced the specificity and avoided immune response during long-term transgene expression [100]. As a major blockage for clinical gene therapy is the occurrence of transgene-specific immunity, which comes from the expression of transgenes in professional antigen-presenting cells [102, 103]. Brown and coworkers exploited the tissue specific expression pattern of miR-142-3p in hematopoietic cells. They placed multiple complementary binding sites to miR-142-3p in the 3’ UTR of the lentivirus vector carrying the gene encoding GFP and challenged the mice with this construct. By this design, the expression of exogenous protein from this construct is supposed to be suppressed in antigen-presenting cells, which has abundant miR-142-3p as hematopoietic cells. Thus, for the mice challenged with the control lentiviral vector lacking the miR-142-3p sites, there were no GFP expression by day 14 due to the immune clearance. In contrast, they observed long-term GFP expression till 120 days post transduction specifically in nonhematopoietic cells, by the lentivirus containing miR-142-3p binding sites. In a separate report, Brown et al further applied this strategy to achieve long-term expression of human clotting factor IX in hemophilia B mice [104]. This expression profile enables stable gene transfer in immunocompetent mice, thus overcoming a major hurdle to successful gene therapy.

As a single cellular miRNA may regulate hundreds of genes, in addition to the miRNA-specific interference, approaches that merely interfere with the regulation of a particular miRNA on a particular gene may also be required for miRNA therapeutic application. Xiao et al explored the possibility of utilizing miRNA’s principle of actions in a gene-specific manner by a miRNA-masking antisense approach [105]. They designed antisense oligodeoxynucleotides (ODN) with locked 5’ and 3’ ends, entirely complementary to the miRNA target motifs in the 3’UTR of cardiac pacemaker channel genes HCN2 and HCN4. The masking antisense ODN can form duplex with the target mRNA to mask the binding site and stop the action of that miRNA. When they were introduced into the cells, these masking molecules remarkably enhanced HCN2/HCN4 expression and their conductance function. Thus, miRNA-masking antisense ODN approach appears to be a valuable supplement to the AMO technique; while AMO is indispensable for the overall function of a miRNA, the miRNA-masking antisense ODN might be more appropriate for the specific outcome of regulation of the target gene by the miRNA.

### 4.3 Replacement of miRNAs

As discussed above, restoring the levels of the altered miRNA expression can be achieved either by using vector overexpressing a specific miRNA or by transient transfection of double-stranded miRNAs. For example, for miRNAs whose expression is downregulated during disease like cancers, restore of the mature miRNA levels in the disease tissue could provide a therapeutic benefit by reinstating the regulation of target gene(s). The synthetic siRNA and cellular mature miRNA duplex actually are functionally interchangeable in terms of *RISC* action against target mRNAs [106]. Therefore, the introduction of double-stranded miRNA duplex mimetic miRNA that is equivalent to the endogenous *Dicer* product and analogous in structure to an siRNA can transiently rescue under-expressed miRNAs. The modifications applied to improve stability and cellular uptake in AMO designs are also practical and useful here, and the benefits conferred by chemical modifications as discussed above have been exhibited experimentally [78, 107, 108]. However, the *in vivo* application of double-stranded miRNA mimics like those conducted for siRNA [88, 109] still awaits evaluation.

To achieve more persistent miRNA replacement, transgene approach is necessary. The specific miRNAs can be enforcedly expressed from a plasmid or viral vector with either polymerase II or III promoter upstream of a short hairpin RNA, which is then processed into mature miRNA by *Dicer* before loading into *RISC*. The most typical approach might be stable expression of miRNA as hairpins by vectors containing pre-miRNA-like shRNAs driven by Pol III promoters such as H1 [110] and U6 [111] promoters. The advantage of these promoters is to provide high expression of miRNAs from well-defined transcription start and termination sites [65]. Recently gene-manipulated mice have been generated overexpressing miR-155, which has been reported to accumulate in human B cell lymphomas [112]. Such miR-155 transgenic mice exhibited a spontaneous B cell malignancy, indicating the oncogenic role of miR155. In a study on cardiac hypertrophy [29], overexpression of miR-133 by adenovirus-mediated delivery of a miRNA expression cassette protected animals from agonist-induced cardiac hypertrophy, whereas reciprocally reduction of miR-133 in wild-type mice by antagomirs caused an increase in hypertrophic markers. Furthermore, instead of designing artificial adapted hairpins, a modification of this approach is to directly clone the entire natural pre-miRNA hairpin into the expression vector [113–117], presuming the endogenous pre-miRNAs are good substrates for Dicer processing. Although a concern may be raised that the expressed pre-miRNA from such vectors may not be processed to release the exact mature miRNA structure without natural flanking sequence to ensure appropriate processing.

High level of the shRNAs from Pol III ensures effective target knockdown; but on the other hand, it may saturate the exportin-5 pathway of endogenous miRNAs leading to off-target effects with fatal consequences [64]. Therefore, in addition to shRNA-Pol III systems, an alternative system that expresses the miRNA in a pri-miRNA form, which includes both the full miRNA flanking sequences and the hairpin structure, shows the great advantage of flexible temporal and spatial control, and this system utilizes Pol II-based inducible and/or tissue-specific expression vectors [65, 80]. Successes of employing this pri-miR-Pol II system have been reported for miR-30 [118] and miR-155 [114]. As in one study, human liver specific miRNA expression vector for miR-122 was constructed, and the tissue specific expression of miR-122 using this vector in HepG2 cells down-regulated the gene expression of HBV [119].

Various studies have further substantiated the potential and flexibility of overexpressing miRNAs with such pri-miR-Pol II transgene system. It is easy to express multiple miRNAs from one transcript by this system. One good example is the miR-155-expressing vector from which multiple same or different miRNAs can be expressed at the same time in a single polycistronic transcript, which resulted in enhanced silencing effect [114]. It was also shown that mRNAs and miRNAs can be coexpressed and functionally act from a single transcript when miRNAs are expressed from the 3’UTR or intron of that gene [114, 118, 120].

### 4.4 miRNA scaffolds

Endogenous pri-miRNAs (or pre-miRNAs) have also been used as scaffolds for the induction of RNA interference, which exhibited the advantage on specificity and efficacy over conventional shRNAs. As aforementioned, a limitation for developing siRNA or shRNA therapy is the possibility of triggering nonspecific interferon responses, which come from the activation of the dsRNA Toll-like receptor TLR3 [121, 122]. Furthermore, another caveat is the competition between the exogenous RNAi triggers like shRNAs and the endogenous miRNAs for the RNAi/miRNA machinery such as *Exportin 5*. Fatality in mice due to oversaturation of cellular miRNA/shRNA pathways was observed [64]. In an evaluation of 49 distinct AAV/shRNA vectors with different designs for six targets, 36 resulted in dose-dependent liver injury, with 23 ultimately causing death due to the downregulation of liver-derived miRNAs. Another study showed that not only exogenous shRNAs but also siRNAs competed with miR-21 in cultured cells by saturating factors besides *Exportin 5* [123]. However, use of endogenous miRNAs as scaffold of RNA interference, in other words, expressing siRNA precursors from the structure of endogenous miRNAs, showed the advantage of overcoming both of the above hurdles. Such artificial miR-siRNA fusion construct can be simply accomplished by replacing sequences in the central stem of endogenous premiRNAs with the siRNA sequences. As in the above study, an exogenous miRNA-based RNAi trigger (or artificial miR-shRNA) with the same siRNA sequence did not display competition with miR-21 [123, 124]. Similarly, it was corroborated by *in vivo* study showing that siRNA expressed from a miRNA backbone did not interfere with endogenous miRNA maturation [125, 126].

To date, the well-defined endogenous miR-26a [123] and miR-30 [113, 116] have been used as typical scaffolds for the expression of RNAi triggers. Note that siRNA expression cassettes based on the precursor of mir-30a and four flanking nucleotides are reportedly effective when transcribed from either Pol II or Pol III promoters[113], even when this hairpin is not a typical Drosha substrate [127, 128]. And a larger flanking region may be necessary for optimal target knockdown by a mir-30-based cassette [117]. Zeng *et al*. [116] have demonstrated that miR-30 miRNA backbone including siRNA sequences effectively degrades mRNAs of endogenous human genes such as the polypyrimidine tract binding protein. McManus et al [123] have demonstrated that siRNA sequences incorporated into the stem of mir-26a function as effectors of RNAi with the specific degradation of homologous mRNA. It has been reported that RNAi triggers contained in pri-miRNA form are processed more efficiently than shRNAs [113], and hairpins with loop sequences derived from pri-miRNAs are more efficiently transported to the cytoplasm than hairpins with artificial loops [129]. Therefore it is not surprising that such miRNA-based RNAi triggers may exhibited much higher silencing efficiency compared to conventional shRNAs [113, 130]. In an antiviral study, 80% more efficiency of gene silencing of HIV-1 p24 antigen using miRNA designed hairpins under U6 promoter than conventional shRNA was shown, supporting miRNA scaffold as a strategy to increase the antiviral potency of RNAi [113]. In a separate study, siRNA expressed from miR-30a scaffold under Pol II promoters achieved similar silencing effect, and a combination of Pol II and Pol III promoters to express two different siRNAs increased the efficacy against HIV-1 replication without comprising cell [131]. Recently, second-generation shRNA libraries covering mouse and human genomes have been designed in the backbone or pri-miR30, showing improvement over the conventional shRNAs [132]. However, it is noteworthy that a contradicting study shows shRNA with 19-nt stem and 9-nt loop may outperform the miR-30 based scaffold for target knockdown in some experimental setting [133].

Besides specificity and efficiency, another important advantage of this RNAi approach is that pri-miRNA scalffolds under Pol II promoters enable the tissue specific and inducible expression. By placing the miRNA scaffold under the control of heat shock-inducible promoter, a miRNA-based conditional RNAi expression system was constructed and it induced conditional gene silencing in response to stress in mammalian cells [134]. By placing miR-shRNA cassette under a tetracycline (Tet)-inducible promoter through Cre-mediated site-specific recombination, a site-directed, virus-free, and inducible RNAi system was developed in embryonic stem cells [120]. Recently, tissue specific and reversible gene silencing *in vivo* has been achieved using the miRNA-based RNAi triggers in transgenic mice [125]. Dickins et al developed transgenic mice harboring a Tet-responsive RNA Pol II promoter-driven miRNA-based RNAi trigger targeting the tumor suppressor Trp53, which reversibly express shRNA when crossed with existing mouse strains expressing general or tissue-specific ‘Tet-on’ or ‘Tet-off’ transactivators. Thus gene knockdown in double-transgenic mice is in a tissue-specific manner, and can be regulated by doxycycline in the drinking water.

Lastly, the miRNA-based RNAi triggers can realize multiple gene knockdowns, which may be necessary to overcome isoform-redundancy in the cell or escape variants during virus infection. A platform using Pol II promoter-driven expression of miRNA-based RNAi triggers which permits robust depletion of multiple target genes from a single transcript has been developed and applied in research on cAMP-dependent transcription [135], and the knockdown efficiency can be enhanced by using multiple mir-shRNA hairpins against the target gene [120].

### 4.5 Artificial miRNAs

With the lax requirement of partial complementarity for targeting, artificial miRNAs can also be designed for a specific set of target mRNAs on demand, which was firstly proposed by Zhang and colleagues [136]. For imbalanced gene expression involving many genes in a disease state, simultaneously regulation of a specific set of genes may be required. Exploiting the miRNA mechanisms of multiple-target regulation, designed artificial miRNAs with the ability to bind to homologous target sites in different mRNAs are potential to execute multiple targeting for the deregulated set of mRNAs in an RNAi-like fashion. For instance, further understanding of miRNA regulation on biological processes would possibly enable us to design artificial miRNAs specific for processes rather than for particular transcripts. In addition, in view of the frequent viral mutations during infection, particularly for RNA viruses, application of artificial miRNAs which work by imperfect complementarity will have immediate advantage on inhibiting mutational escape. However, artificial miRNAs are in its infancy of development. Predictable efficacy and adjustable specificity of artificial miRNAs have been addressed in plants [137–139]. Recently, artificial miRNAs targeting chemokine receptor CXCR4 have been successfully employed to inhibit gene expression in breast cancer cell line [140]. Development of novel algorithm to design highly specific miRNAs for a transcript or group of transcripts has been the current focus [141]. Although certain sequence homologies for miRNA-binding sequences are required for the chosen set of mRNAs, the lenient allowance of miRNA/mRNA mismatches will not make this issue a great limitation. In prospect, computer program-assisted sequence predictions in combination with empirical efforts and more complete knowledge on miRNA mechanisms will promote the development of artificial miRNAs.

## 5. Future Prospect

MiRNA as a novel arm of gene expressional regulation tool has been widely used in gene functional studies of a variety of fields. More importantly, as an emerging technology, it has the great potential to be employed in drug development. Recent advances in the miRNA research have provided us more insights and improved understanding towards miRNA biogenesis, function and particularly their association with molecular pathogenesis of a variety of complex diseases including cancer, heart diseases, chronic viral infections, immune disorders, neurodegenerative disease and metabolic diseases. Currently, the identified oncogenic miRNAs, and viral encoded miRNAs, which are key factors for viral replication and latency, are the ideal targets for developing therapeutics. Moreover, understanding the miRNA signature in susceptible individuals including their expression profiles, dynamics, and even miRNA target variants (single-nucleotide polymorphisms) may eventually enable the miRNA-based individual-specific therapy, as well as disease diagnosis and prognosis. In addition, siRNA/miRNA specific delivery to target cell populations using approaches of nanobiotechnology is just beginning and looks promising. In a word, with the development in miRNA field, these small molecules could be an invaluable tool for various areas of basic and applied research and, more importantly, for therapeutic intervention.
